# Comparative analysis of anesthesia and clinical efficacy of inhalation anesthesia and intravenous anesthesia in Trigeminal Nerve Balloon Avulsion

**DOI:** 10.12669/pjms.39.4.6720

**Published:** 2023

**Authors:** Zhong-jie Zhang, Long-ji Cui, Lin Shen, Xian-bin Ning

**Affiliations:** 1 Zhong-jie Zhang, Department of Anesthesiology, Affiliated Hospital of Beihua University, 132000 Jilin, P.R. China; 2 Long-ji Cui, Department of Pain Clinic, Affiliated Hospital of Beihua University, 132000 Jilin, P.R. China; 3 Lin Shen, Department of Anesthesiology, Affiliated Hospital of Beihua University, 132000 Jilin, P.R. China; 4 Xian-bin Ning Department of Neurosurgery, Affiliated Hospital of Beihua University, 132000 Jilin, P.R. China

**Keywords:** Trigeminal Neuralgia, Inhalation Anesthesia, Intravenous Anesthesia

## Abstract

**Objective:**

To observe the anesthesia and clinical efficacy of inhalation anesthesia and intravenous anesthesia in patients with trigeminal neuralgia undergoing surgery.

**Methods:**

This is a retrospective study. Eighty patients with trigeminal neuralgia admitted to the Affiliated Hospital of Beihua University from July 2018 to July 2021 were selected and divided into two groups according to different anesthesia methods: inhalation group and intravenous group, with 40 cases in each group. Patients in the inhalation group were given inhalation anesthesia with sevoflurane, while those in the intravenous group were given intravenous anesthesia. Hemodynamics, intubation and extubation time, postoperative consciousness recovery, adverse reactions and clinical effects of surgery were compared between the two groups during anesthesia.

**Results:**

During the induction of anesthesia, after induction and after surgery, the levels of hemodynamic parameters in the two groups increased compared with those before induction of anesthesia, and the increase in the inhalation group was smaller (P<0.05). Patients in the inhalation group had a long time from anesthesia to endotracheal intubation but had a short time from completion of surgery to intubation, which was statistically significant compared with the intravenous group (P<0.05). Compared with the intravenous group, the postoperative consciousness recovery time of the inhalation group was significantly shorter and the incidence of adverse reactions was significantly lower (P<0.05).

**Conclusion:**

Inhalation anesthesia with sevoflurane is more effective than intravenous anesthesia in trigeminal neuralgia patients treated with trigeminal nerve balloon avulsion, boasting satisfactory safety, less impact on hemodynamics, and shorter recovery time of consciousness.

## INTRODUCTION

Trigeminal neuralgia (TN) is the most common craniofacial pain, with an annual incidence of 4-13/100,000. It occurs frequently in middle-aged and elderly people, among which the incidence is higher in females than in males, with a male-female prevalence ratio of about 1:1.5-1:1.7.^1^ TN, which is unilateral in most cases, is mainly manifested as short-term paroxysmal, recurrent severe pain in the trigeminal nerve distribution area and is prone to occur when washing face, brushing teeth and daily speaking

It has a serious impact on patients’ psychology, daily life and work, and some patients even suffer from irritability, depression and other mental manifestations, seriously reducing their quality of life.^2^,^3^ Various approaches are clinically available for the treatment of TN, of which oral drug therapy is preferred. Drugs can control most symptoms of TN, but a variety of drug treatment adverse reactions are accompanied, such as dizziness, liver toxicity, inattention, and aplastic anemia. Clinically, many patients choose surgical treatment because of poor efficacy or intolerance to adverse drug reactions.

Percutaneous trigeminal nerve balloon avulsion is a relatively minimally invasive method for the treatment of TN that is widely used in clinical practice, boasting advantages of minimally invasive, safety, and repeatability compared to microvascular decompression and percutaneous radiofrequency thermocoagulation.^4^ Appropriate anesthesia is a prerequisite for successful surgical treatment of TN. Percutaneous trigeminal nerve balloon avulsion is mainly performed under general anesthesia, but the selection of anesthetic drugs directly affects the anesthetic effect and prognosis. In this study, a comparison was conducted between the anesthetic and clinical efficacy of inhalation anesthesia with sevoflurane and combined intravenous anesthesia.

## METHODS

This is a retrospective study. A total of 80 patients with trigeminal neuralgia admitted to the Affiliated Hospital of Beihua University from July 2018 to July 2021 were selected.

### Ethical Approval:

This study was carried out with the approval of the Medical Ethics Committee of our hospital; dated Mar. 11, 2022(No.: JJKH20220077KJ), and all patients were consented of this study.

### Inclusion criteria:

- Patients with clinically diagnosed primary trigeminal neuralgia who underwent percutaneous trigeminal nerve balloon avulsion or reoperation for postoperative recurrence;
- Patients with complete clinical data;
- Aged > 35 years, and aged < 75 years.

### Exclusion criteria:

- Patients with pain caused by other reasons;
- Patients with incomplete clinical data.

All patients were divided into two groups according to different anesthesia methods: inhalation group (40 cases) and intravenous group (40 cases). There were 26 males and 14 females in the inhalation group, aged 40-70 years, with an average of (55.55±8.24) years, and 27 males and 13 females in the intravenous group, aged 41-72 years, with an average of (56.78±8.96) years. No statistically significant difference was observed in the comparison of basic data between the two groups, which were comparable.

### Methods of anesthesia:

Patients were routinely monitored with an electrocardiogram (ECG) in the operating room and intravenous access was established.

### Intravenous group:

Patients were given 0.3μg/kg sufentanil intravenously first, and 3mg/kg propofol intravenously after 2mim. After the loss of consciousness, 0.6 mg/kg of rocuronium bromide was administered intravenously again, and an endotracheal tube was placed on the patients after muscle relaxation to control their breathing.

### Inhalation group:

5% sevoflurane was first inhaled for 3-5 minutes to drain the air in the breathing bag, and then the anesthetic gas was effectively filled into the respiratory loop channel. Subsequently, 0.3μg/kg sufentanil was injected intravenously, and two minutes later, a completely tight sael mask was placed on the face of the patients. Finally, patients were instructed to take a deep breath and inhale sevoflurane at an oxygen flow rate of eight L/minutes. None of the patients underwent muscle relaxation antagonism, and extubation was performed after the patients were fully conscious.

### Observation indicators:

During surgery, ECG monitoring was performed on the patients, and the systolic blood pressure (SBP), diastolic blood pressure (DBP), heart rate (HR) and adverse reactions of the patients were recorded before anesthesia induction (T0), during anesthesia induction (T1), fifteen minutes after anesthesia induction (T2) and after surgery (T3). The time from anesthetics to endotracheal intubation, time from completion of surgery to extubation, postoperative consciousness recovery time, directional force recovery time, first exhaust time and awake time were compared between the two groups. Moreover, the patients were evaluated for surgical efficacy:^5^ the pain relief within seven days after surgery was used as the criterion for short-term efficacy. Excellent: no pain within seven days after surgery; Good: Significant pain relief without the need for analgesic drugs; Poor: No relief in pain. Patients in both groups were followed for one year and their recurrence was recorded.

### Statistical Analysis:

SPSS 22.0 was used for the statistical analysis of all data in this study. Measurement data were expressed as mean ± standard deviation (*χ̅*±*S*), and a *t*-test was used for preoperative and postoperative comparison. Counting data were expressed as n (%), and c² test was used for inter-group comparison. P<0.05 indicates a statistically significant difference.

## RESULTS

At T0, there was no significant difference in the levels of SBP, DBP, and HR between the two groups (all P>0.05); At T1, T2, and T3, the levels of SBP, DBP, and HR between the two groups were increased compared with those at T0, and the levels of SBP, DBP, and HR in the inhalation group at T1, T2, and T3 were lower than those in the intravenous group, with statistically significant differences (all P<0.05), as shown in Table-I.

**Table-I:** Hemodynamic indicators of the two groups (*χ̅*±*S*).

Time	Inhalation group			Intravenous group		

	SBP (mmHg)	DBP (mmHg)	HR (time/min)	SBP (mmHg)	DBP (mmHg)	HR (time/min)
T_0_	112.78±3.13	78.63±2.12	84.20±4.88	113.23±2.88	79.05±2.29	84.08±6.01
T_1_	122.73±3.08*	86.65±2.08*	93.90±4.61*	126.90±2.48	90.10±2.32	96.43±5.47
T_2_	119.85±3.14*	84.55±2.07*	90.20±4.75*	129.20±2.81	91.85±2.40	98.03±5.70
T_3_	114.85±3.13*	78.98±2.01*	83.30±4.69*	117.58±3.43	79.95±2.14	85.90±5.93

***Note:***

* P<0.05 compared with the intravenous group.

Patients in the inhalation group had a long time from anesthesia to endotracheal intubation but had a short time from completion of surgery to extubation, which was statistically significant compared with the intravenous group (all *P*<0.05). The consciousness recovery time, directional force recovery time, first exhaust time and awake time of the inhalation group were shorter than those of the intravenous group, with statistically significant differences (all *P*<0.05), as shown in Table-II.

**Table-II:** Anesthesia indicators of the two groups (*χ̅*±*S*).

Item	Inhalation group	Intravenous group	t value	P value
Time from anesthesia to endotracheal intubation (min)	5.38±0.70	4.83±0.71	3.472	0.001
Time from completion of surgery to extubation (min)	9.88±1.18	12.75±1.28	10.459	0.000
Consciousness recovery time (min)	11.38±1.92	14.45±2.19	6.673	0.000
Directional force recovery time (min)	20.75±2.25	27.45±1.52	15.610	0.000
First exhaust time (h)	29.50±2.24	38.60±1.50	21.343	0.000
Awake time (min)	18.75±1.79	23.40±1.57	12.352	0.000

Bradycardia, pruritus, dizziness, nausea and vomiting occurred in both groups, but the incidence of adverse reactions in the inhalation group was significantly lower than that in the control group, with a statistically significant difference (P<0.05), as shown in Table-III.

**Table-III:** Occurrence of adverse reactions in the two groups [cases (%)].

Group	Bradycardia	Pruritus	Dizziness	Nausea and vomiting	Adverse reaction rate
Inhalation group (n=40)	2 (5.00)	1 (2.50)	3 (7.50)	2 (5.00)	8 (20.00)
Intravenous group (n=40)	3 (7.50)	4 (10.00)	6 (15.00)	4 (10.00)	17 (42.50)
c^2^ value					4.713
P value					0.030

An excellent and good rate was obtained in both groups after surgery. There were three cases of recurrence in the inhalation group and five cases in the intravenous group. No statistically significant difference was observed in the comparison of the excellent and good rate as well as recurrence rate between the two groups (all *P*>0.05), as shown in Table-IV.

**Table-IV:** Surgical effects of the two groups [cases (%)].

Group	Excellent	Good	Poor	Excellent and good rate	Recurrence rate
Inhalation group (n=40)	28 (70.00)	9 (22.50)	3 (7.50)	37 (92.50)	3 (7.50)
Intravenous group (n=40)	26 (65.00)	8 (20.00)	6 (15.00)	34 (85.00)	5 (12.50)
c^2^ value				1.127	0.556
P value				0.288	0.456

## DISCUSSION

Trigeminal neuralgia (TN) is severe electrocute-like pain in the innervation area of one or more branches of the trigeminal nerve, lasting from a few seconds to a few minutes.^6^ A variety of clinical methods are currently available for the treatment of TN. In the wake of the improvement of technology and the emergence of new materials in recent years, percutaneous trigeminal nerve balloon avulsion has gradually been widely used.^7^ It has become the preferred method for the treatment of TN due to its advantages of simple operation, short learning period, minimally invasive, rapid and efficient, short hospital stay and low cost. This approach is particularly well suited for those with trigeminal neuralgia who are unable or unwilling to undergo craniotomy and have severe underlying diseases. In view of individual differences of patients, different anesthesia methods have different effects on patients’ treatment and stress response of the body. Studies have shown^8^,^9^ that ideal anesthesia can reduce the sensitization of the central nervous system, relieve the body’s stress response to pain, be conducive to the stability of the internal environment, and provide satisfactory conditions for patients to recover during and after surgery.

During surgery, an ideal anesthesia plan must be one that has a fast onset, controllable depth of anesthesia, stable vital signs, and quick recovery of patients after surgery. In the past, intravenous anesthesia with ketamine or propofol was often used. Ketamine anesthesia was administered intravenously, with fast onset and favorable analgesic effect, but long-term administration is required to ensure the anesthetic effect; Intravenous anesthesia with propofol has the advantages of quick onset, strong and stable effect, short duration, fewer adverse reactions and quick recovery. However, it has a strong inhibitory effect on the circulatory system and affects the activity of sympathetic nerves.^10^ Moreover, intravenous anesthesia with propofol has high requirements for anesthesiologists and is difficult to be popularized in hospitals, especially in primary hospitals. In the past, ether was often used for inhalation anesthesia. However, ether had an unpleasant smell, and patients were prone to adverse reactions such as irritability, nausea, and dizziness. Sevoflurane is a new colorless and transparent inhalation anesthetic that can reduce airway irritation and stabilize the anesthesia process. It has the following characteristics:

- Little airway stimulation, stable and rapid anesthesia induction and recovery;
- Little effect on the nervous system;
- No obvious inhibitory effect on the respiratory system;
- Little effect on the circulatory system without causing increased catecholamine concentration;
- Short half-life, rapid recovery and induction, and satisfactory analgesic effect.^11^-^14^

This study showed that for patients with trigeminal neuralgia treated by trigeminal balloon avulsion, sevoflurane inhalation anesthesia was safe, had little effect on hemodynamics, and the recovery time of consciousness was short.

As shown in the results of this study, there was no significant difference in surgical effect between the inhalation group and the intravenous group. The recurrence rate of anesthesia patients in the inhalation group was 7.5%, while that in the intravenous group was 12.5%, showing no statistically significant difference. Both anesthesia methods can achieve favorable anesthesia effect and enable surgeons to complete the operation smoothly, with satisfactory surgical efficacy, indicating that both methods are safe and reliable in clinical application. Adverse reactions occurred in both anesthesia methods during anesthesia, and the incidence of adverse reactions in the inhalation group was significantly lower than that in the intravenous group (20.00% vs 42.5%), with a statistically significant difference (P<0.05). It is suggested that inhalation anesthesia with sevoflurane can significantly reduce the occurrence of adverse reactions during anesthesia owing to the characteristics mentioned above.

It was shown in this study that at T1, T2, and T3 after anesthesia, the SBP, DBP and HR of the two groups were all increased compared with T0, but the increase in the inhalation group was lower than that in the intravenous group, indicating that propofol anesthesia had a significant impact on SBP, DBP and HR of the patients. Inhalation anesthesia with sevoflurane is superior to intravenous anesthesia with propofol in that it has a better anesthetic effect, lower effect on hemodynamics, higher efficacy and safety. Dhande et al.^15^ pointed out that sevoflurane maintains better hemodynamic stability compared to propofol, and patient acceptance of both drugs is similar. However, if induced by sevoflurane for a long time with high concentration, patients are prone to lower blood pressure. To this end, the inhalation concentration of sevoflurane should be adjusted in time after 2-3 minutes of inhalation, and sufentanil should be injected at the same time, which can reduce tracheal intubation response, but also reduce unconscious limb movement; moreover, the combined use of these two drugs boasts a synergistic effect that reduces cardiovascular stress responses and decreases circulatory inhibition.^16^ According to Li J et al.^17^, administration of sufentanil after induction of anesthesia reduced emergence agitation in children receiving sevoflurane anesthesia for adenotonsillectomy compared with fentanyl. It was shown in our study that the recovery time of consciousness, directional force recovery time, first exhaust time and postoperative awake time of patients in the inhalation group were faster than those in the intravenous group, indicating that inhalation anesthesia with sevoflurane has an ideal sedation effect, shorter duration of anesthesia and faster postoperative recovery of consciousness.^18^,^19^

### Limitations of this study:

It includes the number of subjects included in this study was limited, so the conclusions drawn may not be very convincing. In addition, we only analyzed and discussed the cases included in our hospital, which may not be representative enough. We look forward to a multi-center study in the future to reach more comprehensive conclusions.

## CONCLUSION

Inhalation anesthesia with sevoflurane is more effective than intravenous anesthesia in trigeminal neuralgia patients treated with trigeminal nerve balloon avulsion, boasting satisfactory safety, less impact on hemodynamics, and shorter recovery time of consciousness.

### Authors’ Contributions:

**ZZ and XN** designed this study, prepared this manuscript, are responsible and accountable for the accuracy and integrity of the work.

**LC** collected and analyzed clinical data.

**LS** Data analysis**,** significantly revised this manuscript.
